# Roles of JAK2 in Aging, Inflammation, Hematopoiesis and Malignant Transformation

**DOI:** 10.3390/cells8080854

**Published:** 2019-08-08

**Authors:** Florian Perner, Caroline Perner, Thomas Ernst, Florian H. Heidel

**Affiliations:** 1 Innere Medizin 2, Hämatologie und Onkologie, Universitätsklinikum Jena, 07747 Jena, Germany; 2 Leibniz-Institute on Aging–Fritz Lipmann Institute (FLI), 07745 Jena, Germany; 3 Dana-Farber Cancer Institute, Department of Pediatric Oncology, Harvard University, Boston, MA 02467, USA; 4 Center for Immunology & Inflammatory Diseases, Massachusetts General Hospital, and Harvard Medical School, Boston, MA 02129, USA

**Keywords:** JAK2, Janus-kinase, aging, clonal hematopoiesis (CHIP), myeloproliferative neoplasia (MPN)

## Abstract

Clonal alterations in hematopoietic cells occur during aging and are often associated with the establishment of a subclinical inflammatory environment. Several age-related conditions and diseases may be initiated or promoted by these alterations. JAK2 mutations are among the most frequently mutated genes in blood cells during aging. The most common mutation within the JAK2 gene is JAK2-V617F that leads to constitutive activation of the kinase and thereby aberrant engagement of downstream signaling pathways. JAK2 mutations can act as central drivers of myeloproliferative neoplasia, a pre-leukemic and age-related malignancy. Likewise, hyperactive JAK-signaling is a hallmark of immune diseases and critically influences inflammation, coagulation and thrombosis. In this review we aim to summarize the current knowledge on JAK2 in clonal hematopoiesis during aging, the role of JAK-signaling in inflammation and lymphocyte biology and JAK2 function in age-related diseases and malignant transformation.

## 1. Development of Clonal Hematopoiesis During Aging

Normal hematopoietic stem cells (HSCs) show intact self-renewal and regeneration with a balanced differentiation potential towards myeloid and lymphoid progenitor cells. As the hematopoietic system ages, somatic mutations lead to a decline in the HSC function and skewing towards the myeloid compartment [1,2]. The first evidence supporting the idea that the hematopoietic system is subject to substantial clonal alterations during aging was provided by studies detecting an age-associated skewing of X-chromosome inactivation patterns in the blood cells of healthy females, particularly in the myeloid compartment [3,4]. The subsequent detection of age-related *TET2* mutations in healthy individuals with X-inactivation skewing and hence clonal hematopoiesis provided one of the first proofs that somatic genetic aberrations occur in the healthy aging population and are associated with an age-related myeloid lineage bias. Furthermore, the detected mutations had been previously observed in patients with myeloid malignancies, suggesting that these clones may represent a premalignant state [5].

More recently, three large exome-analysis studies have shown age-related clonal hematopoiesis in healthy individuals, driven by mutations of genes recurrently mutated in myeloid neoplasms and associated with an increased risk of hematologic cancer and cardiovascular disease. Whilst healthy individuals younger than 50 years of age were rarely affected (<1%), about 10% of individuals over 65 years of age showed clonal hematopoiesis. All studies identified similar genes, with the majority of mutations affecting epigenetic modifiers such as *DNMT3A*, *TET2* and *ASXL1*. Among other frequently mutated genes were those involved in RNA-splicing such as *SF3B1* and *SRSF2*, and genes involved in signal transduction such as *CBL* and *JAK2* [6,7,8]. Based on these findings, Steensma et al. [9] proposed the term clonal hematopoiesis of indeterminate potential (CHIP) for individuals who do not show conclusive morphologic and clinical evidence of malignant or pre-malignant disease, but harbor somatic mutations frequently detected in hematologic malignancies.

**Figure 1:**
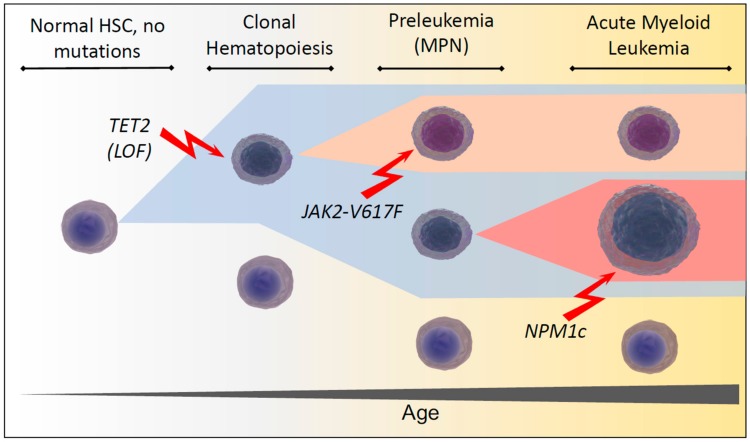
Schematic of clonal development in hematopoietic stem and progenitor cells during aging. Representative example of the sequence of TET2 loss of function (LOF), Janus Kinase 2 (JAK2-V617F) and Nucleophosmin (NPM1c) mutations leading to clonal hematopoiesis, a myeloproliferative neoplasia (MPN) and finally to the development of acute myeloid leukemia (AML) respectively.

Interestingly, Young et al. have since used ultra-sensitive DNA-sequencing to demonstrate persisting leukemia-associated mutations at very low frequencies in 95% of healthy individuals aged 50 to 60 [10], suggesting that age-related clonal hematopoiesis is extremely common, if not inevitable. A prime example of the apparent paradox of healthy individuals carrying aberrations associated with myeloid malignancies, is the *JAK2-V617F* mutation, which has been considered to be the major culprit in causing polycythemia vera (PV) and other myeloproliferative neoplasms (MPN) for over a decade [11,12,13,14]. Yet recently it was shown that *JAK2-V617F* neoplasms develop from clonal hematopoiesis over many years with highly variable levels of clonal expansion [15]. Taken together these findings raise questions about the cascades of events necessary to transform clonal hematopoiesis from a seemingly common and benign to a relatively rare malignant state (Figure 1).

## 2. Consequences of Deregulated JAK2 Signaling in Hematopoiesis and Aging, CHIP-Driven Inflammation and Consequences of JAK2-Mutations

Even though the complex functions of age-related clonal alterations are just about to be explored by the scientific community one common feature of several described CHIP-mutations is the establishment of a sub-clinical inflammatory state. In addition to JAK2-mutations, particularly loss-of-function of TET2 has been described to be associated with chronic proinflammatory changes in vivo [16,17,18,19]. Of note, these indolent inflammatory alterations may become clinically apparent upon additional stress factors like infections. In 2018, Meisel et al. described the interplay between the TET2 loss-of-function and microbial stimuli to drive pre-leukemic myeloproliferation using a Tet2−/− knockout mouse model [20]. DNMT3A, the most frequently mutated gene in clonal hematopoiesis, has been identified as a critical mediator of mast cell responses [21]. Furthermore, also other genes that are frequently mutated in CHIP, like SF3B1 [22], CBL [23,24], GNB1 [25] and GNAS [26] have been recently explored as crucial mediators of inflammatory signals. Among the genes that are common subjects to age-related mutations JAK2 has been linked most clearly to inflammatory processes since it serves as a signal transmitter downstream of major cytokine receptors [27,28,29]. Therefore, multiple inflammatory conditions, including those driven by other genes mutated in CHIP, converge on the JAK signal transduction and are dependent on the JAK-activity. Type I and II cytokine receptors are a conserved family of transmembrane proteins including the receptors for interleukins, interferons, erythropoietin, thrombopoietin, growth hormone, leptin and colony stimulating factors (CSFs) [30,31]. These cytokine receptors lack an intrinsic kinase activity but are associated with “Janus kinases” (JAKs). The JAK-family consists of four members: JAK1, JAK2, JAK3 and TYK2 that have different association patterns to receptors [32,33,34,35,36,37]. JAK1 and JAK2 have non-redundant functions and are essential during development. Therefore, respective conventional knockout mouse models exhibit postnatal or embryonic lethality, respectively [38]. In the JAK2 knockout mice a complete absence of definitive erythropoiesis and interferon-gamma insensitivity was observed causing lethality at day 12.5 postcoitum [38]. The conventional JAK1 knockout allowed normal tissue development but caused postnatal lethality due to nursing insufficiency. In these animals the different interleukin and interferon responses were dramatically impaired [39]. Accordingly, inactivating germline mutations in JAK1 or JAK2 cannot be found in humans. Loss of the JAK3 function on the other hand is vital and causes a severe combined immunodeficiency (SCID) syndrome in patients and germline mutations of TYK2 can be found in patients and are a cause of an autosomal dominant form of the hyper-IgE syndrome [40].

**Figure 2:**
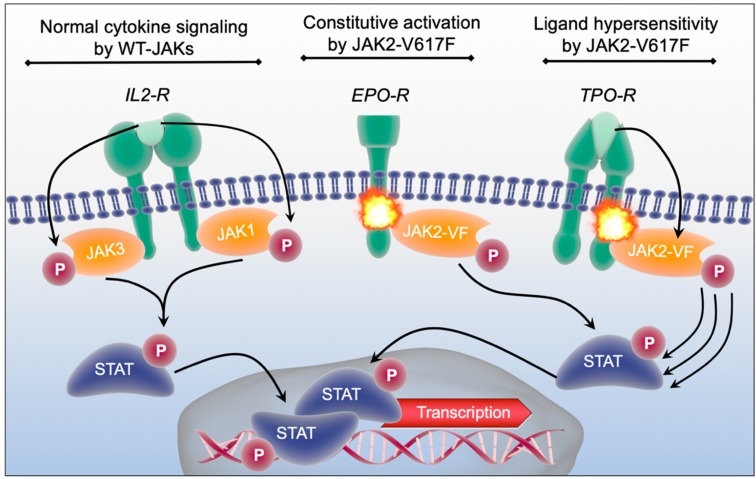
Schematic representation of normal and aberrant JAK-signal transduction. The IL2-receptor (IL2-R) associated with JAK1 and JAK3 serves in this cartoon as an example for physiological JAK-signaling dependent on cytokine binding. JAK2-V617F (JAK2-VF) is representatively depicted in association with the Erythropoetin-receptor (EPO-R) and the Thrombopoetin-receptor (TPO-R) where it induces constitutive activation and ligand hypersensitivity, respectively.

Following activation of the cytokine receptors through binding of the respective ligand JAKs become phosphorylated and promote phosphorylation and activation of “Signal Transducers and Activators of Transcription” (STATs) (Figure 2, left scheme) [41,42,43,44,45]. STATs are DNA-binding proteins that contain seven members (STAT1, STAT2, STAT3, STAT4, STAT5a, STAT5b, STAT6). After dimerization of STATs through activation, they translocate from the membrane to the nucleus to regulate the transcription of target genes (Figure 2, bottom) [42,44]. In addition to the STATs, JAK downstream signaling is believed to be mainly executed by the “Mitogen-activated protein kinases” (MAPK) as well as the “Protein kinase B pathway” (PI3K/ AKT). Accordingly, JAKs can promote proliferation, differentiation and cytokine production in immune- and hematopoietic stem and progenitor cells [46,47]. These pathways can be pathologically activated by either excessive ligand binding (e.g., under chronic inflammatory conditions) or by activating mutations within JAK-genes. The JAK2-V617F point mutation is the most frequently detected somatic mutation in the JAK family that leads to constitutive activation of JAK2 and its downstream effectors independent from ligand availability as well as to a hypersensitivity of cytokine receptors upon ligand binding (Figure 2, middle and right schemes) [12,48]. Thereby, JAK2-V617F is a frequent driver of myeloproliferative neoplasms (MPN), a group of myeloid malignancies caused by an increased proliferation and cytokine production of different myeloid cell types [11,12,13,14]. Due to their involvement in multiple inflammatory pathways, JAKs are attractive targets for therapeutic intervention in inflammatory diseases [49,50,51,52]. Here, JAK-inhibition can lead to a reduction of symptom burden in MPN [53,54] as well as in different rheumatological diseases [49,55,56,57].

### 2.1. Dysfunctional JAK-Signaling and JAK-Inhibition in the Adaptive and Innate Immune System

As described in more detail above, JAKs have a crucial function in cytokine-driven signal transduction and are therefore central molecules in the immune system. Given the fact that cytokine signals induce differentiation and polarization of leukocyte subpopulations one can anticipate that alterations in the JAK activity and function may have profound consequences for white blood cell homeostasis. The activating JAK2-V617F mutation that drives MPN and is also detected in individuals with CHIP can indeed influence lymphocyte biology. In 2017, Nishanth et al. reported on a gain-of-function phenotype induced by mutations of JAK2 in T-cells [58]. In MPN the JAK2-V617F mutation arises from mutated HSC clones and is therefore not strictly committed to myeloid cells. The authors reported on 6/13 MPN patients harboring a JAK2-V617F mutation in a significant percentage of their CD4+ and CD8+ T-cells. Using a knock-in model of JAK2-V617F [59] with an inducible T-cell specific CD4-Cre-recombinase animals showed improved pathogen clearance during Listeria monocytogenes infection with increased cellular and serological responses. Otherwise, the thymic T-cell development was not significantly impaired. In contrast, increased numbers of neutrophils and erythroblasts could be detected in the spleens indicating a cell extrinsic effect of the JAK2-V617F+ T-cells on myeloid bystanders through cytokine secretion [58]. This experimental model provided first insights into how activating JAK2 mutations influence the lymphocyte function and confirmed the complexity of the interplay between the cell intrinsic and humoral effects of JAK2-V617F. In other reports Stijnis et al. assessed for the JAK2-V617F mutation in different lymphocyte subsets of MPN patients with co-existing chronic lymphocytic leukemia (B-CLL). The authors described JAK2-mutations in T-cells, NK-cells and polyclonal B-cells, but not within the monoclonal B-cell population (CD19+ CD5+ B-CLL-cells) [60]. Most notably, recent reports provided first evidence on how the JAK2-V617F mutated myeloid cells may influence T-cell responses. JAK2-V617F promoted the synthesis of PD-L1 in MPN cells leading to limited anti-neoplastic T-cell responses, metabolic changes in T-cells and eventually JAK2-V617F-driven immune-escape of MPN cells [61]. These findings may facilitate the use of immunotherapeutic approaches for JAK-mutated clones. The question how far the presence of JAK2-mutations in distinct lymphocyte subsets predict differential responses or outcomes in MPN patients or individuals with CHIP has not been addressed yet.

The consequences of JAK-inhibition on human immune cell function have been studied in more detail. In early clinical trials, increased numbers of viral infections have been described in MPN patients on treatment with the JAK1/2 inhibitor ruxolitinib [53,62]. JAK1/2 inhibition in MPN patients leads to a reduction in CD3+ T-cells and decreased cytokine production. Within the T-cell compartment regulatory T-cells (Treg) and Th1 cells seem to be most prominently affected [63,64,65]. Additionally, effector functions of CD8+ T-cells are impaired upon JAK1/2 inhibitor treatment [66]. Furthermore, JAK-inhibition compromises B-cell differentiation and antibody production [67,68] as well as dendritic cell function [69]. Even though the spectrum of the JAK-inhibition is broad, the suppression of cellular functions is cell type specific and incomplete. Therefore JAK-inhibition is relatively well tolerated by patients in randomized clinical trials [53,70,71,72] with a relatively low rate of infectious complications. Accordingly, JAK-inhibitor should be considered an immunomodulatory treatment rather than a classical immunosuppressive drug. In addition to the already mentioned attempts to utilize JAK-inhibitors in rheumatological diseases they are successfully used in the treatment of acute and chronic graft-versus-host disease (GvHD) after allogenic stem cell transplantation. In a mouse model of GvHD ruxolitinib improved survival and limited proinflammatory cytokine production as well as Th1 and Th17 polarization. A pilot trial of ruxolitinib in steroid refractory GvHD patients successfully limited cytokine production and clinical symptoms in this heavily pre-treated cohort [73]. Furthermore, advanced clinical trials investigating ruxolitinib for GvHD in a randomized fashion are currently under way. Interestingly, the graft-versus-leukemia effect which is required for the therapeutic efficacy remained unaffected [74], confirming the model of selective immunomodulatory functions of JAK. In how far different JAKs have redundant and non-redundant functions in immune cells is yet not fully understood. Since ruxolitinib is currently the only approved JAK inhibitor for the treatment of MPN but novel and more specific compounds are being developed, the question whether specificity of inhibitors for the JAK1 or JAK2 function (or both) are required for the T-cell function [75] is of utmost importance. We utilized two JAK1/2 inhibitors that are in clinical use (ruxolitinib and momelotinib) as well as a novel specific JAK2-inhibitor (BSK805). Interestingly, in vitro treatment of healthy donor T-cells with either JAK1/2 inhibitor resulted in the inhibition of proliferation, global activation (CD69), and STAT1 phosphorylation of CD4+ and CD8+ T-cells while selective JAK2-inhibition had no such effect. To confirm these findings we genetically inactivated either JAK1 or JAK2 in T-cells using RNAi. Only JAK1 depletion was sufficient to inhibit the global T-cell function in vitro. Consistently, JAK2 was dispensable for global T-cell effector functions in vivo in a mouse model of GvHD [75]. These findings highlight the importance of JAK-selectivity depending on the underlying condition or context. Selective JAK2 inhibitors are currently in clinical trials [76,77,78,79,80] and seem to be equally effective in symptom control and inhibition of systemic inflammation. The interplay between the different JAKs may limit the applicability of highly selective JAK-inhibitors [81]. Of note also JAK1, JAK3 and TYK2 selective inhibitors are currently under investigation in clinical trials for different inflammatory diseases [82,83,84,85,86,87,88,89,90,91,92,93].

### 2.2. Chronic Inflammation and JAK Signaling in Aging and Age-Related Degenerative Diseases

Aging and age-related diseases are accompanied by sterile, low grade and chronic inflammatory processes in different tissues [94,95,96,97,98,99,100,101]. Even though unbalanced inflammation during aging may lead to tissue degeneration and age related diseases, temporal and spatial orchestration of inflammatory stimuli is crucial for tissue homeostasis and regeneration [102,103,104,105,106,107]. In particular the importance of the JAK/STAT pathway for regenerative processes in multiple tissues has been highlighted [108,109,110,111,112,113]. In 2016, Shen et al. reported on their findings from a longitudinal study with 91 young and older individuals profiling immunological signatures and cytokine responsiveness over the course of three years. The authors found elevated baseline levels of activated STATs, most prominently in T-cells, leading to a reduced responsiveness to cytokine stimulation in the cohort of elderly individuals. Of note these changes where associated with cardiovascular diseases [114]. Based on these observations it is tempting to speculate in how far the chronic inflammatory state established by age related clonal mutations may participate to these changes in systemic homeostasis during aging.

Several studies investigated JAK-function and -inhibition in different degenerative aging related conditions. With regards to the issue of age-related cachexia one study using a respective mouse model pointed out that excessive activation of the JAK-STAT pathway in aged adipose tissue is inducing a senescence-associated secretory phenotype (SASP) with increased production of proinflammatory cytokines. Moreover, administration of a JAK1/2 inhibitor to aged mice for 10 weeks reduced inflammation and alleviated frailty [115]. Decreased or diminished regenerative capacity of skeletal muscle is another age-related symptom that can be challenged through pharmacological inhibition of JAK2 or STAT3 by stimulating symmetric expansion of satellite cells in vitro and their engraftment in vivo. Intramuscular injection of JAK-inhibitors resulted in a remarkable enhancement of muscle repair and regeneration [116]. In Parkinson´s and Alzheimer´s disease alpha-synuclein and Amyloid-beta accumulation is leading to activation and pre-sensitizing of microglia through the JAK/STAT pathway, resulting in an IFN-γ driven proinflammatory microglial phenotype promoting neurodegeneration. Treatment with JAK inhibitors prevented the degeneration of dopaminergic neurons in an in vivo model of Parkinson´s disease and attenuated the IFN-γ-induced changes in cultured microglia and in isolated microglia prepared from APP/PS1 Alzheimer´s mice [117,118]. In how far kinase inhibition could be utilized as an intervention for age related functional decline or chronic degenerative diseases is currently a subject to controversial discussions within the community [119,120]. For manifesting clinical conditions like neurodegenerative diseases some of those treatment approaches may enter clinical trials in the future due to the lack of alternative therapeutics and tolerability of potential side effects. For subclinical conditions like muscle or adipose tissue loss dietary and lifestyle interventions should primarily be considered to impact on health outcomes.

### 2.3. Pathophysiology of JAK2 in Malignant Transformation and Myeloproliferative Neoplasia

While JAK2-mutations are among the most common genetic aberrations detected in aging-associated clonal hematopoiesis they do not necessarily lead to **development of myeloproliferative neoplasms** or hematologic cancers [6,7,121,122]. Nevertheless, activating JAK2-V617F mutations are the most frequent driver mutations of myeloproliferative neoplasms (MPN) [123,124]. The vast majority of these activating JAK2 mutations are JAK2-V617F point mutations as mentioned above [14]. Furthermore, there is a small proportion of JAK2-V617F-negative MPN patients showing alterations in Exon 12 of the JAK2 gene leading to a similar disease phenotype [125]. Studies on primary human blood specimens had reported on the presence of other gene mutations before the acquisition of JAK2 mutations and raised the question whether the JAK2-mutation alone is sufficient for malignant transformation [126,127,128,129]. Most recently, however, elegant studies performed in murine models provided first evidence that in principle a single hematopoietic stem cell can be transformed by an activating JAK2-V617F-mutation and lead to erythrocytosis or thrombocytosis in vivo [130]. The presence of the JAK2-V617F-mutation in HSCs accelerated cell division and resulted in increased DNA damage, however, the penetrance of disease initiation in mouse models was low. Longitudinal studies focusing on the evolution of mutations in serial human samples found a low mutation rate of one mutation per 66 patient years [131] arguing against a strong hypermutable state in MPN. Of note, the strongest predictor of disease progression and outcome in these analyses was the number of somatic mutations that occurred in addition to driver mutations as JAK2. Therefore, individuals that exclusively harbor JAK2-mutations in the hematopoietic system, may experience long-term stability of the mutated clone or even clonal regression. In contrast, individuals with more than one mutation have a significantly higher risk of clonal expansion and progression [132,133,134]. The presence of clonal aberrations prior to the acquisition of the JAK2-V617F-mutation may therefore provide a ‘fertile ground’ for malignant transformation [131,135]. Once the clone has expanded and results in myeloproliferation, the presence, type and order of clonal aberrations will influence the disease phenotype and prognosis [136,137,138,139]. Although clinical markers are established to assess for prognosis of phenotypically defined subtypes of MPN, genomic classification can identify patients with common biologic factors that accurately define patients at risk for disease progression and further expansion of the malignant clone [140]. Consistent with these findings, mutations in genes such as TP53, TET2 or IDH1 have been frequently observed in primary samples of patients who had experienced transformation of MPN into blast phase or secondary acute myeloid leukemia [141,142,143] and suggest a role of these secondary events in leukemic transformation.

Although the dynamics of clonal expansion and mutational landscape have been defined in very detail, several aspects of disease development, maintenance and progression remain so far elusive. One unique aspect of JAK-V617F-mutations is the low dependency of mutated clones on the constitutive activity of the kinase. While in other cancers, inactivation of oncogenic kinases or receptors results in rapid and durable regression of the malignant clone [144,145], pharmacologic inhibition of JAK2 does not affect the overall disease burden or evolution of persistent clones to a major extent [146,147]. Combination with inhibitors of cell signaling or epigenetic compounds may facilitate targeting of the malignant clone. Likewise, the effects of extrinsic factors, such as inflammatory stimuli caused by co-morbidities or repeated infections dynamics on the JAK-mutated clones are incompletely understood.

**The inflammatory phenotype** that is observed in MPN patients is a major aspect of JAK2-induced pathophysiology and contributes to morbidity and mortality of the disease [28]. As outlined above in more detail, Janus-kinases are critical mediators of cytokine, chemokine and growth-hormone signaling. Constitutive activation of Janus-kinase due to mutations in the JAK-gene locus therefore results in inflammation that can promote myeloproliferation, thrombosis formation, and splenomegaly and is the major cause of constitutional symptoms [148,149] in patients with MPN. Even similarities of inflammatory symptoms and laboratory parameters with other reactive causes of inflammation (e.g., systemic inflammatory response syndrome–SIRS) have been described [28]. In murine models of MPN as well as in primary patient samples, high levels of pro-inflammatory cytokines could be detected [150,151,152,153]. Moreover, high levels of cytokine expression have been described to be prognostically relevant [152]. As one prominent example, tumor necrosis factor alpha (TNFa) was found to be highly expressed in JAK2-V617F-mutated MPN cells and to correlate with disease burden in primary patient cells [154]. Inactivation of TNFa in murine models of MPN or primary patient cells resulted in reduced disease development in vivo and abrogation of clonal growth, respectively. Recent publications provided first evidence that besides the JAK2-mutated clone also non-malignant ‘bystander’ cells can contribute to the secretion of pro-inflammatory cytokines [155]. Cytokine profiles of the JAK-mutated clone are depending on STAT-signaling and can partially overlap with those of non-malignant ‘bystanders’ or those produced by other driver mutations (e.g., thrombopoietin-receptor mutations). Secretion of pro-inflammatory cytokines and chemokines also results in tissue re-modeling in the bone marrow, specifically in the development of fibrosis and osteosclerosis. Moreover, the inflammatory milieu may by itself promote myeloproliferation and clonal evolution [27]. One model system that supports this hypothesis is overexpression of the transcription factor NF-E2, which has shown to be highly expressed in primary MPN samples [156,157,158]. The resulting inflammatory milieu promoted cellular proliferation and disease progression.

Anti-inflammatory compounds have been used to treat JAK2-V617F-driven MPN since decades. The early use of corticosteroids in patients with myelofibrosis was followed by the use of interferons, IMIDs and most recently JAK-inhibitors [123]. JAK-inhibitors have shown significant clinical activity on the reduction of inflammation [54,71]. Clinical use of JAK-inhibitors has shown to improve elevated levels of pro-inflammatory cytokines and associated symptoms such as weight loss, fever or splenomegaly.

Mutations of JAK2 have been linked with **thromboembolic complications** and alterations in hemostasis and cell adhesion in vitro and in vivo [159]. Several pathophysiologic aspects contribute to this thrombophilic state: (i) Hypercellularity induced increase in blood viscosity, (ii) JAK-induced alterations in plasmatic coagulation and vessel wall, (iii) JAK-induced changes of cell adhesion and function. Early observations reported increased rates of thromboembolic events in patients with myeloproliferative neoplasia [160,161]. Along these lines, control of hypercellularity (such as hematocrit control below 45% in polycythemia vera) results in reduction of thromboembolic complications. This therapeutic goal is achieved irrespective of the type of cytoreduction, through recurrent phlebotomies, pharmacologic cytoreduction using hydroxycarbamide or a combination of both [162]. Therefore, the cytoreductive treatment is a mainstay of the MPN therapy to prevent thromboembolic complications, especially in high risk situations defined by higher age or previous thromboembolic events [163]. However, although impairment of blood flow due to hypercellularity is an obvious risk factor for thrombus formation and development of arterial emboli, even patients in early phases of myeloproliferative neoplasia and with normal blood counts develop thrombotic events, e.g., of the splanchnic venous system [164]. Furthermore, the presence of cardiovascular risk factors increases the risk for thromboembolic complications in JAK2-V617F-mutated MPNs [165,166,167]. This indicates that other factors such as plasmatic coagulation or cell intrinsic alterations of JAK-mutated cells may contribute to thrombus formation. Most recently, murine models of MPN have given insight into JAK-induced pathophysiology regarding thrombotic complications. Expression of mutated JAK2 in endothelial cells (a finding that could be confirmed in the human system; [168,169,170]) resulted in abnormalities in blood flow and alteration of plasmatic coagulation [171]. Moreover, high expression of mutated JAK2 was associated with both, formation of unstable thrombi and bleeding events in vivo [171,172]. JAK-mutated platelets showed reduced activatability and moderate glycoprotein (GP) VI deficiency while a decreased proportion of high-molecular-weight von-Willebrand-factor multimers could reduce platelet adhesion [172]. Consistently, activation of JAK2-V617F-mutations in megakaryopoiesis resulted in hypersensitivity to thrombopoietin (Tpo) stimulation, higher mobility of megakaryocytes, elevated pro-platelet formation and increased thrombus formation due to enhanced platelet aggregation [173]. These findings are consistent with the clinical situation where JAK2-mutations indicate an increased risk for thromboembolic events per se compared to non-JAK-mutated MPN cases [174] and administration of salicylic acid is routinely used to prevent thrombotic complications [175]. When focusing on red blood cell (RBC) biology, other groups could show that abnormal adhesion of JAK2-V617F-mutated red blood cells is induced through upregulation adhesion molecules through Rap-Akt-signaling even in the absence of erythropoietin (Epo) [176]. In this model system, pharmacologic inhibition of Rap- or Akt-signaling was able to reduce abnormal adhesion of RBCs. In neutrophil biology, JAK2-V617F-mutations seem to affect various cellular functions. Primary patient neutrophils harboring activating JAK2-mutations show a significant increase in neutrophil extracellular trap (NET) formation [177]. Likewise, murine JAK2-V617F-mutated neutrophils contributed to increased thrombus formation following experimental venous ligation. Pharmacologic JAK-inhibitor treatment effectively reduced the phenotypes in both model systems. Of note, an extended clinical and molecular analysis on a large cohort of otherwise healthy individuals with clonal hematopoiesis (CHIP) revealed significantly higher rates of thromboembolic events in those harboring JAK2-mutations as clonal markers when compared to non-JAK-mutated CHIP [177]. Consistent with these findings, increased expression of beta1-integrins was described on granulocytes of MPN patients harboring JAK2-mutations as compared to age-matched healthy donor controls [178]. Functional studies using mouse models and primary human cells confirmed activation of integrins by JAK2 in a Rap1-GTPase dependent manner [179]. The activation of integrins may also contribute to the accumulation of myeloid cells in the spleen, as pharmacologic inhibition of the integrin function in mice resulted in improvement of splenomegaly and reduced cell numbers in the spleens. These results may facilitate a combination of Janus-kinase inhibitors with inhibitors of integrin activation or function to efficiently prevent thrombosis formation patients at risk.

## 3. Conclusions

Age-related somatic mutations in hematopoietic stem cells lead to the establishment of clonal sub-populations in the blood of elderly individuals and is frequently associated with chronic inflammatory changes. The presence of these clonal alterations is associated with cardiovascular risk and malignant transformation. Among the most frequently mutated genes during aging is the Janus kinase JAK2. The activating JAK2-V617F mutation is a driver of myeloproliferative neoplasia (MPN) a preleukemic blood disorder. Aberrant activation of JAK2 in MPN is associated with hyperproliferation of myeloid progenitor cells, abnormal inflammatory cytokine release as well as hyper-agglutination and thrombosis. The role of JAK mutations in clonal hematopoiesis of indeterminate potential (CHIP) on the other hand remains largely elusive. Of note even in the absence of a clinically manifest MPN JAK2-V617F appears to be associated with thromboembolic complications. Since most major cytokine and growth factor receptors utilize JAKs to transmit their signals this group of kinases form a common intersection in inflammatory signal transduction on where multiple cytokine driven responses converge. Therefore, pharmacological targeting of JAKs can serve as a therapeutic tool to modulate the activity of a broad range of immune functions. Importantly, inhibitors of JAKs are in clinical use for the treatment of MPN and graft compared to the host disease (GvHD) as well as for several rheumatological diseases, including rheumatoid arthritis, psoriasis and lupus erythematosus. Chronic inflammatory processes negatively influence on health outcomes during aging. One driver of this chronic sterile inflammation is the aberrant cytokine release and the respective responses of cells in different organs. Since JAKs transmit most of those signals they are centrally involved in mediating this alteration in systemic homeostasis. Recent evidence suggests that several age-associated functional alterations are driven or promoted by this chronic inflammation. Indeed, the decline in muscle, adipose tissue and neuronal function during aging can partially be challenged by the mediating JAK activity. Nevertheless, in how far drug treatment of these chronic age-related conditions may be beneficial in certain cases remains controversial.
